# High-Resolution OCT of Basal Laminar Deposit Phenotypes and Retinal Pigment Epithelium Morphologic Changes in Age-Related Macular Degeneration

**DOI:** 10.1016/j.xops.2026.101415

**Published:** 2026-09-23

**Authors:** Alberto Quarta, Shinichiro Chujo, Paolo Forte, Alessandro Feo, Jianfeng Huang, Rouzbeh Abbasgholizadeh, Federico Sivilotti, Ceren Soylu, Seungmin Lee, Giulia Corradetti, David Sarraf, Srinivas R. Sadda

**Affiliations:** 1 Doheny Eye Institute, Pasadena, California; 2 Stein Eye Institute, Department of Ophthalmology, David Geffen School of Medicine, University of California, Los Angeles, California; 3 Department of Ophthalmology, Mie University Graduate School of Medicine, Mie, Japan; 4 Ophthalmology Unit, Fondazione IRCCS Ca' Granda Ospedale Maggiore Policlinico, Milan, Italy; 5 Beijing Hospital, National Center of Gerontology, Institute of Geriatric Medicine, Chinese Academy of Medical Sciences, Beijing, P.R. China; 6 Eye Clinic, Department of Biomedical and Clinical Science “Luigi Sacco”, Sacco Hospital, University of Milan, Milan, Italy; 7 Ophthalmology Department, Pusan National University School of Medicine, Yangsan, South Korea

**Keywords:** Age-related macular degeneration, Basal laminar deposit, Retinal pigmented epithelium, Subretinal drusenoid deposit

## Abstract

**Purpose:**

To characterize the high-resolution OCT phenotype of basal laminar deposit (BLamD) and the codistribution of retinal pigment epithelium (RPE) morphologic changes in patients with age-related macular degeneration (AMD).

**Design:**

Cross-sectional observational study.

**Participants:**

Thirty-one eyes from 19 patients (10 female, 9 male; mean age 85.6 ± 5.5 years) with AMD and OCT-visible BLamD.

**Methods:**

Structural high-resolution OCT B-scans were systematically graded for BLamD phenotype (persistent, scalloped, and outer retinal corrugations), RPE morphologic changes (spiky RPE and intraretinal hyperreflective foci [IHRF]), foveal involvement, and additional features (hypocore drusen, subretinal drusenoid deposits [SDDs], and soft drusen), including geographic atrophy (GA), complete RPE and outer retinal atrophy (cRORA), and incomplete RPE and outer retinal atrophy.

**Main Outcome Measures:**

Prevalence and co-occurrence of BLamD and RPE phenotypic features stratified by foveal status.

**Results:**

Of the 117 screened patients with AMD, 19 (16.2%) had OCT-visible BLamD, contributing 31 eyes: 28 with cRORA, 1 with incomplete RPE and outer retinal atrophy, and 2 without OCT-defined atrophy. Persistent BLamD was the most prevalent finding (28/31, 90.3%), followed by scalloped BLamD and IHRF (both 21/31, 67.7%) and spiky RPE (18/31, 58.1%). All 31 eyes exhibited ≥1 RPE–BLamD complex abnormality, and 27/31 eyes (87.1%) demonstrated ≥2 concurrent features. Outer retinal corrugations were identified in 4/31 eyes (12.9%) and were exclusively present in eyes without spiky RPE (4/13, 30.8% vs. 0/18, 0%). Foveal-spared GA eyes showed higher prevalences of spiky RPE (9/14, 64.3% vs. 6/14, 42.9%) and IHRF (11/14, 78.6% vs. 7/14, 50.0%) compared with foveal-involved eyes. Eyes lacking persistent BLamD (3/31, 9.7%) paradoxically showed all spiky RPE, scalloped BLamD, and IHRF. Additional findings included hypocore drusen in 8/31 eyes (25.8%), SDD in 31/31 eyes (100%), and soft drusen in 4/31 eyes (12.9%).

**Conclusions:**

In BLamD-associated GA, persistent and scalloped BLamD, spiky RPE, IHRF, and outer retinal corrugations showed variable patterns of co-occurrence. Spiky RPE and IHRF were more frequent in foveal-sparing than in foveal-involved eyes, although the significance of this observation requires longitudinal validation. These findings support the integration of BLamD morphologic subtyping and RPE phenotyping into clinical and trial-based assessments of advanced AMD, with potential implications for patient selection and monitoring in the era of approved complement inhibition therapies.

**Financial Disclosure(s):**

Proprietary or commercial disclosure may be found in the Footnotes and Disclosures at the end of this article.

Age-related macular degeneration (AMD) is a complex degenerative disease of the outer retina in which extracellular deposits are central to clinical classification, disease staging, and progression.^1^, ^2^, ^3^ These deposits include drusen, basal linear deposit, subretinal drusenoid deposit (SDD), and basal laminar deposit (BLamD), each occupying distinct anatomic compartments and likely reflecting partially distinct pathways of retinal pigment epithelium (RPE), Bruch's membrane (BrM), photoreceptor, and choriocapillaris degeneration.^1^^,^^3^, ^4^, ^5^, ^6^ Among them, BLamD has long been recognized as a histopathologic feature of AMD, although early descriptions often considered it within the broader context of aging change, early maculopathy, and sub-RPE extracellular deposition rather than as a distinct in vivo imaging phenotype.^1^^,^^4^^,^^7^, ^8^, ^9^

The importance of BLamD was further clarified by Sarks et al,^3^^,^^10^^,^^11^ who correlated BLamD and membranous debris with the clinical phenotype of early AMD and showed that increasing deposit burden parallels progressive RPE degeneration and the pathway toward geographic atrophy (GA). Importantly, Sarks et al^3^^,^^11^ also distinguished early and late forms of BLamD, emphasizing that BLamD is not a uniform lesion but an evolving substrate that changes with disease progression.

Recent histology–OCT correlation studies have renewed interest in BLamD as a clinically detectable structure rather than a purely microscopic finding.^4^^,^^12^^,^^13^ Sura et al^4^ showed that thickening of the RPE–basal lamina–BrM complex in AMD is driven largely by BLamD rather than by expansion of either the RPE or BrM.

With axial resolution approaching 3 μm, high-resolution OCT allows improved visualization of subtle internal variations within the RPE–basal lamina–BrM compartment.^14^, ^15^, ^16^, ^17^, ^18^ Our group recently used high-resolution OCT to characterize BLamD–RPE complex abnormalities in eyes with extensive macular atrophy with pseudodrusen-like appearance (EMAP), showing that persistent and scalloped BLamD phenotypes frequently coexist with RPE irregularity, intraretinal hyperreflective foci (IHRF), and SDD overlying BLamD.^19^ Recognizing different morphologic expressions of BLamD may have implications for AMD phenotyping, interpretation of atrophy margins, and analysis of disease progression.^3^^,^^4^^,^^12^^,^^20^^,^^21^

The aim of this study is therefore to characterize BLamD-related phenotypes in eyes with AMD using 3-μm axial resolution high-resolution OCT. Specifically, we sought to describe the morphologic spectrum of OCT-visible BLamD and evaluate associated OCT features, including scalloped configuration, persistent BLamD, and RPE alterations.

## Methods

### Study Design and Participants

This observational cross-sectional study included consecutive patients with AMD. The study was approved by the institutional ethics committees of the University of California, Los Angeles and was conducted in accordance with the Declaration of Helsinki and applicable national privacy regulations, including the Health Insurance Portability and Accountability Act. Subjects were consecutively recruited from the Doheny-University of California, Los Angeles Eye Centers (Pasadena, CA) between January 2023 and April 2026.

Eyes were eligible for inclusion if they met the following criteria: (1) a definitive diagnosis of AMD confirmed through comprehensive clinical evaluation and multimodal imaging according to Beckman criteria^22^; (2) clear ocular media without high refractive error (within ±6 diopters); (3) availability of high-resolution OCT volumes acquired with automatic real-time averaging ≥20 frames; (4) absence of motion or segmentation artifacts; (5) high image quality with signal strength index >20 dB; and (6) visible BLamD.^19^ The exclusion criteria included: (1) any ocular or systemic disorder that could confound image interpretation or alter retinal structure including glaucoma, foveal hypoplasia, or diabetic retinopathy; (2) prior intraocular surgery other than uncomplicated phacoemulsification performed ≥6 months before enrollment; (3) clinical or historical evidence of macular neovascularization or prior anti-VEGF therapy; (4) detection of nonexudative macular neovascularization on a customized en face swept-source OCT angiography slab encompassing the RPE–BrM complex, corroborated by corresponding flow signal on coregistered structural OCT; and (5) coexisting vitreoretinal interface abnormalities, such as epiretinal membrane or vitreomacular traction, that could distort retinal anatomy or impact visual function.

### Study Population and Procedures

All participants underwent a comprehensive ophthalmic evaluation including a detailed medical and ocular history, best-corrected visual acuity testing, intraocular pressure measurement, slit lamp biomicroscopy, and ultra-widefield fundus imaging (Optos California RGB or Silverstone RGB; Optos). Multimodal imaging also included high-resolution OCT (Spectralis, Heidelberg Engineering GmbH), blue-light and green-light fundus autofluorescence when available, and 6 × 6-mm swept-source OCT angiography scans centered on the fovea acquired using the PLEX Elite 9000 platform (Carl Zeiss Meditec).

The high-resolution OCT is an investigational device developed by Heidelberg Engineering GmbH that provides an axial resolution of 3 μm in tissue, compared to the 7 μm of the standard SPECTRALIS HRA + OCT system. This improvement is achieved by shifting the central wavelength from 880 to 853 nm and broadening the spectral bandwidth from 50 to 137 nm, while maintaining a lateral resolution of approximately 14 μm. To compensate for the reduction in signal-to-noise ratio associated with the increased bandwidth, the incident optical power was increased from 1.2 to 2.2 mW. The system employs a superluminescent diode light source; ocular exposure has been evaluated by the manufacturer in accordance with applicable international safety standards (ANSI Z80.36 and IEC 60825-1) and remains within established limits for clinical OCT imaging.

High-resolution OCT volumes were acquired using a 20° × 20° or 30° × 25° raster protocol with automatic real-time averaging ≥25 frames, 97 and or 121 B-scans per volume, and signal-to-noise ratio >20. The minimum requirement for the study was 97 B-scans. Geographic atrophy on OCT was identified according to the Classification of Atrophy Meeting consensus definition for incomplete and complete RPE and outer retinal atrophy (cRORA).^23^ Diagnosis of AMD with GA was established by 2 independent masked graders (C.S and A.Q.), with adjudication by a senior grader (S.S.) in cases of disagreement, on the basis of comprehensive multimodal imaging review and in the absence of imaging findings suggestive of alternative macular disorders.

### High-Resolution OCT Assessment of BLamD and RPE

Graders were masked to clinical data and to all other imaging modalities. Each volume was qualitatively evaluated for proper foveal centration and overall image quality; scans demonstrating motion artifacts, poor signal strength, or misalignment of the retinal layers were excluded. A given feature, assessed within 500 μm from the cRORA or incomplete RPE and outer retinal atrophy border, was recorded as present when it appeared on ≥2 consecutive B-scans within the same anatomic region, defined according to the ETDRS ring subfield system. Given the descriptive design of the study, features were characterized qualitatively and recorded as present or absent; no ordinal severity grading was applied. OCT features were defined on the basis of previously published clinicopathologic correlation studies.^4^^,^^8^^,^^24^, ^25^, ^26^, ^27^

• Thin BLamD was defined as a smooth single hyperreflective band located between the RPE and BrM. On high-resolution OCT, this deposit appears narrow and nonlaminated, without internal stratification or splitting of the RPE–BrM complex. This phenotype corresponds to early BLamD as described in AMD clinicopathologic studies and may represent an earlier stage of sub-RPE matrix accumulation prior to lamination.^1^^,^^4^
• Scalloped BLamD was defined as a compact and multilaminated deposit between the RPE and BrM. On high-resolution OCT, this appears as ≥1 discrete hyporeflective laminae, with >1 reflective interface, and a characteristically concave-outward scalloped outer contour. This organized morphology corresponds to late BLamD in AMD histopathology.^3^^,^^4^^,^^7^
• Persistent BLamD was identified as an amorphous and nonlaminated accumulation of material at the level of BrM without a continuous overlying RPE layer, typically confined to regions of cRORA. On high-resolution OCT, this material has a granular and structureless appearance without clear lamination, corresponding to the residual epi-BrM BLamD seen in AMD after RPE loss and representing the end-stage of the BLamD continuum.^3^^,^^4^^,^^28^^,^^29^

Thin BLamD was considered a background age-/disease-associated finding expected across the study eyes and was therefore not evaluated as a separate morphologic category. The analysis focused on persistent and scalloped BLamD, which represented the principal BLamD patterns assessed in relation to overlying RPE alterations (Figure 1, Figure 2, Figure 3, Figure 4, Figure 5).

**Figure 1:**
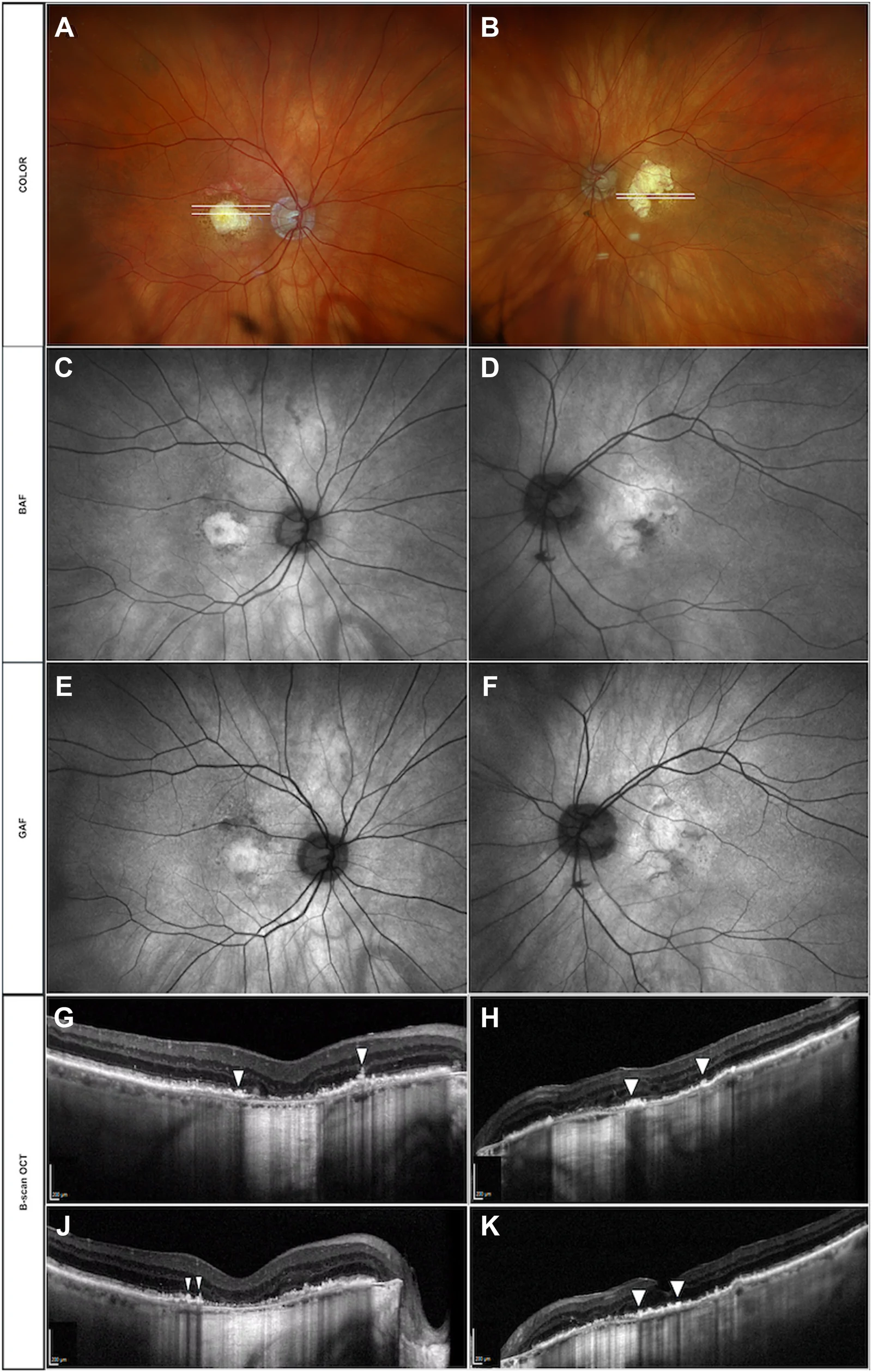
Multimodal imaging of BLamD in GA secondary to age-related macular degeneration, with associated spiky RPE on high-resolution OCT. Color fundus photographs of the left and right columns (**A, B**) show GA with surrounding pigmentary alteration and corresponding scan locations. Blue-light autofluorescence (**C, D**) demonstrates heterogeneous hypoautofluorescence within the lesion, with a surrounding rim of relatively increased autofluorescence. Green-light autofluorescence (**E, F**) shows the same lesions with altered contrast and border conspicuity, highlighting the complementary information provided by the 2 autofluorescence modalities. The high-resolution OCT B-scans (**G**, **H, J**, and **K**) disclose abnormalities at the level of the RPE–basal lamina–Bruch's membrane complex adjacent to and within the atrophy. In the upper B-scans (**G, H**), arrowheads indicate focal spiky excrescences arising from the RPE band. These lesions appear as small, sharply hyperreflective, vertically oriented projections extending from an irregular RPE layer into the overlying outer retina. Their morphology is distinct from subretinal drusenoid deposits, as they originate within the RPE, share its high reflectivity, and are associated with local distortion of the normally smooth RPE contour. In the lower B-scans (**J, K**), the arrowheads identify more conspicuous spiky RPE changes at the atrophy margin. Here the RPE is no longer perceived as a simple continuous monolayer, but as an irregular and dysmorphic band with focal anterior projections, suggestive of RPE activation, hypertrophy, and early anterior migration. These spiky excrescences overlie a diseased sub-RPE compartment characterized by BLamD and are seen in association with attenuation or loss of outer retinal bands. BAF = blue autofluorescence; BLamD = basal laminar deposit; GA = geographic atrophy; GAF = green autofluorescence; RPE = retinal pigment epithelium.

**Figure 2:**
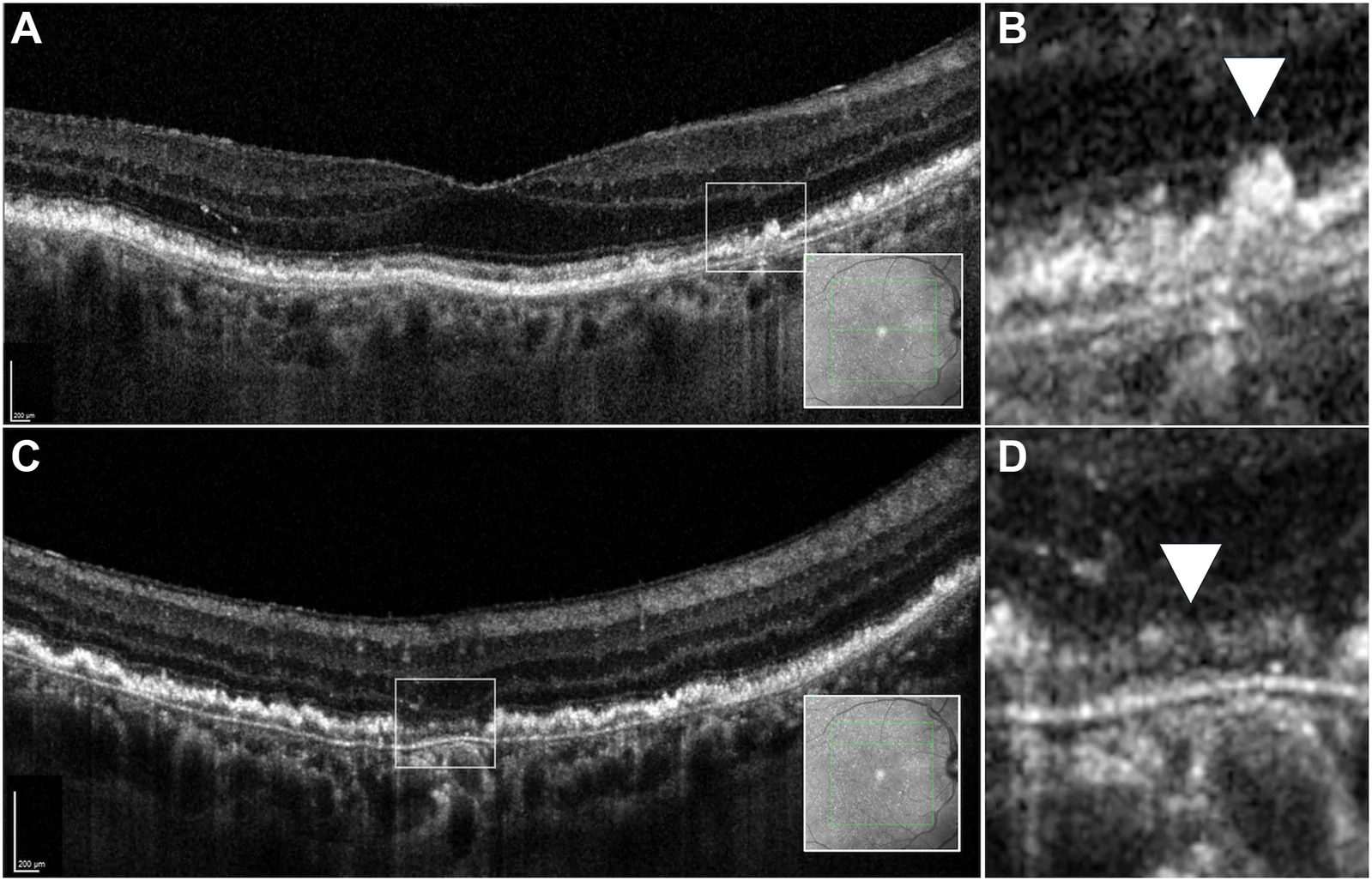
High-resolution OCT examples of spiky RPE in eyes with age-related macular degeneration. **A** and **C**, High-resolution OCT B-scans through regions of outer retinal and sub-RPE abnormality. Insets indicate the scan position, and the boxed areas are enlarged in panels **B** and **D**. In both examples, the arrowheads identify focal spiky excrescences arising from the RPE layer. The lesions appear as discrete, sharply hyperreflective, apically directed projections that emanate from an otherwise irregular RPE band and extend into the overlying outer retina. In the upper row (**A, B**), the spiky lesion is solitary and compact. The projection arises from the RPE surface, shares the reflectivity of the parent RPE band, and protrudes anteriorly into the subretinal space. In the lower row (**C, D**), the RPE band is more diffusely irregular, with a crenulated contour and multiple small elevations along the RPE–basal lamina–Bruch's membrane complex. Within this background, the arrowheaded lesion represents persistent BLamD. BLamD = basal laminar deposit; RPE = retinal pigment epithelium.

**Figure 3:**
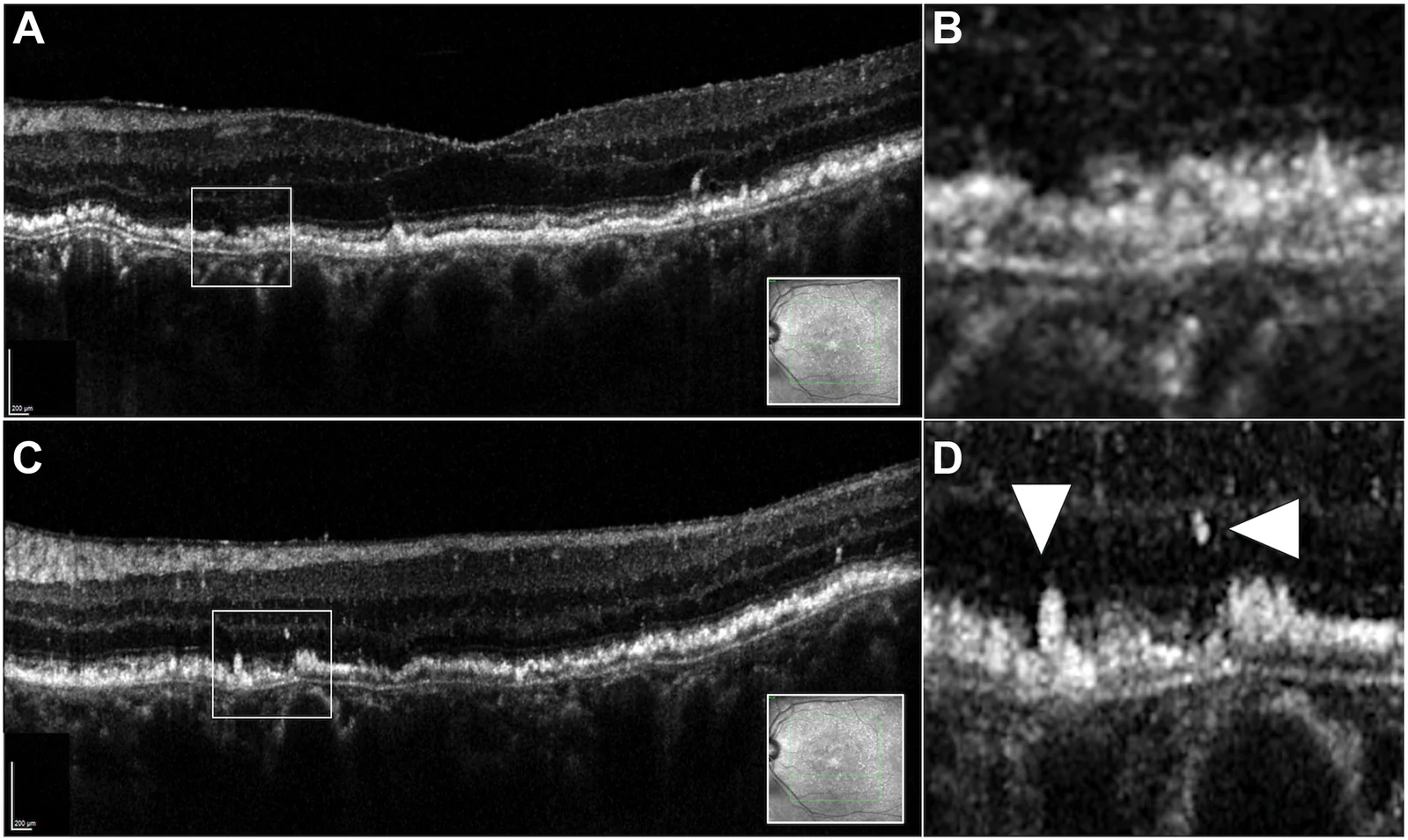
High-resolution OCT of spiky RPE with focal anterior migration. High-resolution OCT B-scans (**A, C**) show localized abnormalities of the RPE–basal lamina–Bruch's membrane complex in eyes with AMD. Boxed regions are enlarged in panels **B** and **D**. In the upper row (**A, B**), the RPE band is irregular and locally thickened, with small apical excrescences projecting into the overlying outer retina. These projections arise from the RPE layer itself and impart a serrated or uneven inner contour to the RPE surface. In the lower row (**C, D**), the same process is more conspicuous. The arrowheads in panel **D** identify 2 hyperreflective foci extending anteriorly from the RPE layer into the outer retina. These lesions are narrow, vertically oriented, and similar in reflectivity to the parent RPE band, supporting their interpretation as dysmorphic RPE rather than subretinal material. AMD = age-related macular degeneration; RPE = retinal pigment epithelium.

**Figure 4:**
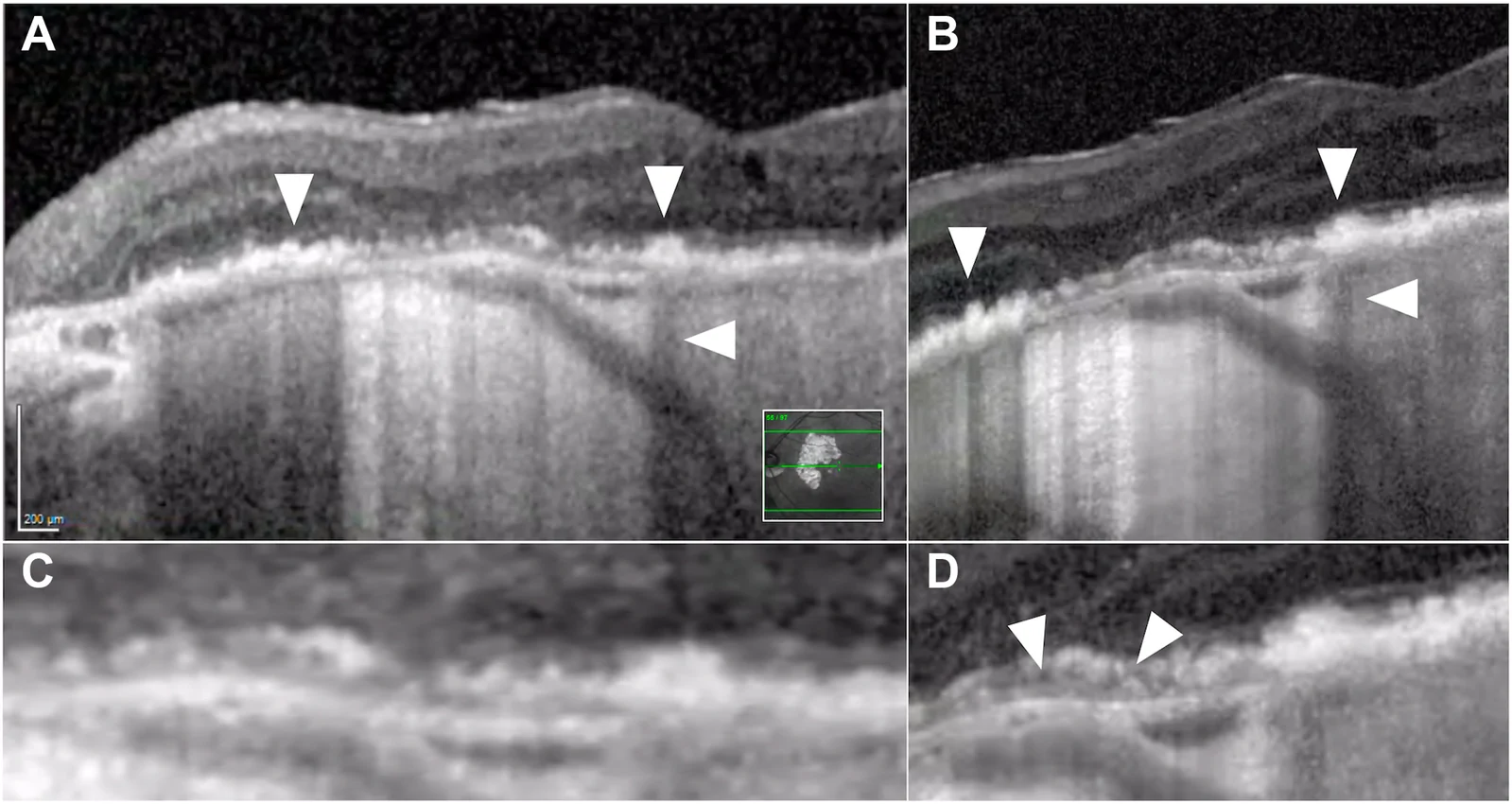
Standard and high-resolution OCT features of persistent BLamD. **A**, Standard Spectralis OCT B-scan through an area of non-neovascular age-related macular degeneration shows an irregular, hyperreflective RPE–basal lamina band with pointed or “spiky” elevations of the RPE (arrowheads). Persistent BLamD is visible as a hyperreflective, undulating line external to the retina and internal to Bruch's membrane, with associated choroidal hypertransmission beneath areas of RPE attenuation. Arrowheads indicate spiky RPE/BLamD and a column of increased signal transmission into the choroid. **B,** High-resolution OCT through the same lesion confirms the same structural signatures with improved axial detail: sharp RPE irregularities, persistent BLamD overlying Bruch membrane, and focal hypertransmission corresponding to loss or attenuation of overlying RPE. Arrowheads mark the spiky RPE and hypertransmission. **C,** Magnified view of (**A**) demonstrates the poor distinguishable laminar organization of the persistent BLamD, appearing as a thin, hyperreflective, corrugated layer distinct from deeper Bruch's membrane/choroidal reflectivity. **D,** Enlarged view of (**D**), the lesion margin highlights focal, tooth-like RPE peaks and adjacent hypertransmission, illustrating how high-resolution OCT resolves the same abnormalities seen on standard Spectralis particularly for persistent BLamD. BLamD = basal laminar deposit; RPE = retinal pigment epithelium.

**Figure 5:**
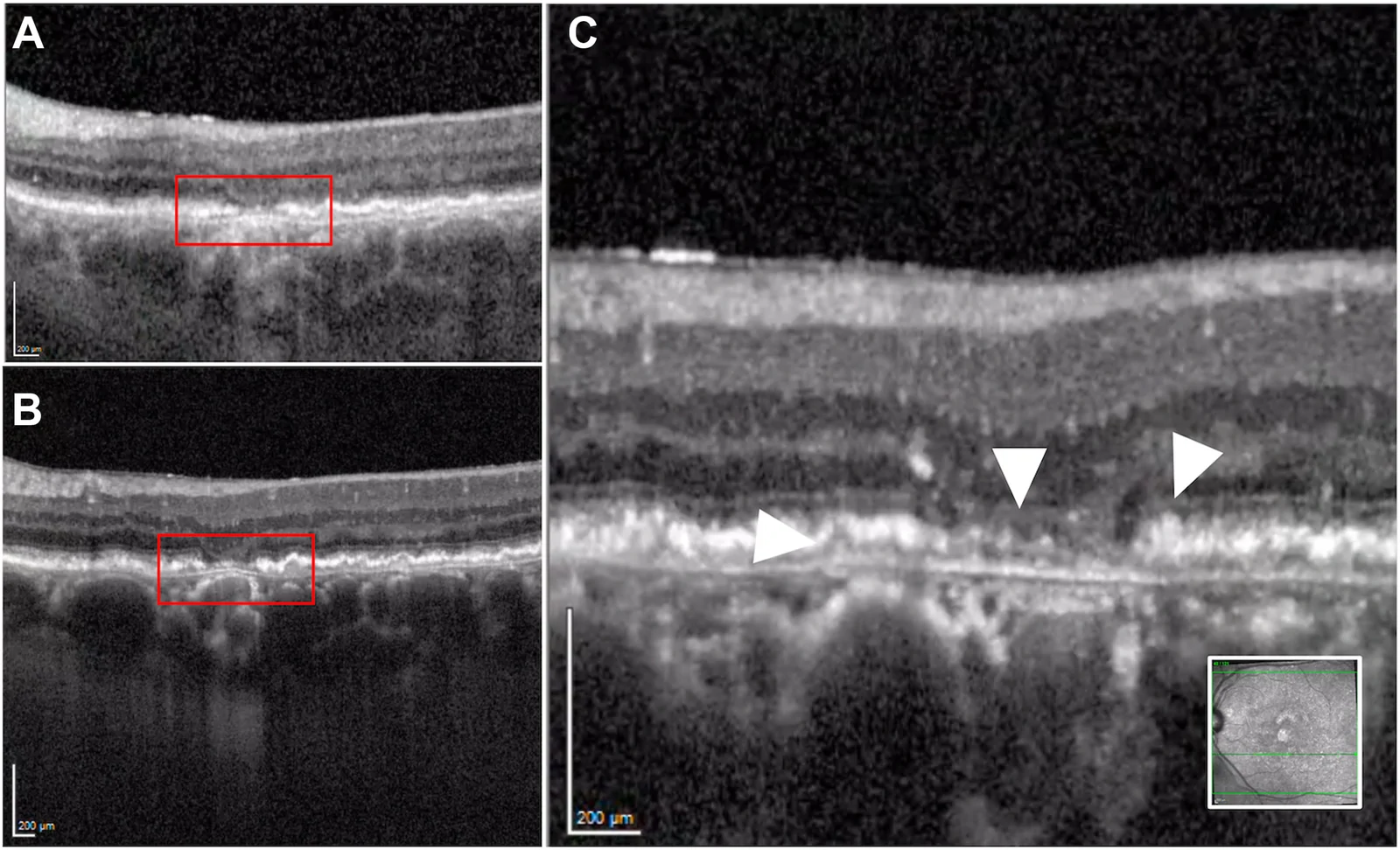
Standard Spectralis and high-resolution OCT of persistent BLamD with RPE dysmorphia and hypertransmission. **A,** Standard Spectralis OCT B-scan shows a shallow, irregular hyperreflective band at the level of the RPE–basal lamina/Bruch's membrane complex. The boxed region contains subtle RPE/BLamD irregularity and focal signal transmission into the choroid. **B,** High-resolution OCT through the same region shows the lesion with improved contrast and axial definition. The boxed region demonstrates a corrugated, hyperreflective RPE/BLamD complex with focal spiky RPE elevations and subjacent choroidal hypertransmission. **C,** Magnified high-resolution inset of the boxed region in **B** highlights the persistent BLamD as a hyperreflective, undulating layer above Bruch's membrane and focal RPE (arrowheads). BLamD = basal laminar deposit; RPE = retinal pigment epithelium.

Retinal pigment epithelium morphologic changes were defined as any deviation from a smooth, continuous, uniformly hyperreflective RPE band, including irregular thickening, apparent multilayering, or spike-like apical protrusions of the inner surface of the RPE layer.^19^ These features were graded in reference to the histopathologic continuum of RPE phenotypes described in the Project MACULA classification in association with BLamD development.^29^^,^^30^ On OCT, RPE abnormalities or spiky RPE were recognized as irregular, thickened, or multilayered reflectivity or nodular/spiky protrusions of the inner surface of the RPE. In contrast, absence of spikes was indicated by an RPE band that was smooth, uninterrupted, uniformly hyperreflective, and without protrusions. Subretinal drusenoid deposits were distinguished from spiky RPE based on their subretinal location, lower reflectivity relative to the RPE band, and absence of posterior shadowing, whereas spiky RPE originated within the RPE layer, exhibited RPE-level hyperreflectivity with frequent shadowing, and demonstrated vertical, columnar morphology consistent with early RPE migration.^19^

Intraretinal hyperreflective foci were defined as discrete, well-circumscribed, punctate hyperreflective lesions ≥3 pixels in size within the neurosensory retina, with reflectivity exceeding that of the retinal nerve fiber layer and approximating that of the RPE, as previously described.^31^, ^32^, ^33^, ^34^, ^35^ Foveal-sparing cRORA was defined and graded similar to previous studies.^36^^,^^37^ Subretinal drusenoid deposits were identified according to the criteria of Zweifel et al.^38^ On structural OCT, soft drusen were defined as dome-shaped elevations of the RPE over BrM, containing predominantly homogeneous, low-to-medium reflective material within the sub-RPE space.^39^^,^^40^ Drusen with a hyporeflective core were defined as drusen showing internal hyporeflective or heterogeneous material, typically associated with a hyperreflective cap, intralesional hyperreflective foci or dots, and, when present, posterior choroidal hypotransmission.^41^ Outer retinal corrugations or coils were defined according to previous definition.^12^ Every feature was assessed by 2 independent masked graders (C.S. and A.Q.), with adjudication by a senior grader (S.S.).

### Statistical Analysis

Statistical analysis was performed on a per-eye basis and was limited to descriptive methods. Categorical variables are presented as counts and percentages, and continuous variables as mean ± standard deviation. The frequency of each OCT-derived morphologic feature was first calculated for the overall cohort. Eyes were then stratified by the presence or absence of selected findings, including scalloped BLamD, persistent BLamD, RPE morphologic changes, and foveal involvement, and the distribution of the remaining OCT features was summarized within each subgroup. Because the study was descriptive, no inferential analyses or hypothesis testing were performed. All analyses were conducted using R version 4.1.0 (R Foundation for Statistical Computing).

## Results

### Study Population

In the screened AMD cohort, visible BLamD was identified in 19/117 patients (16.2%). A total of 31 eyes from the 19 patients (10 female, 9 male; mean age, 85.6 ± 5.5 years; range, 69–96 years, median 86 years; interquartile range, 84–87) were included after exclusion of 4 eyes with concurrent neovascularization (Table 1, Supplementary material 1, available at www.ophthalmologyscience.org). Both eyes were included for 12 participants, yielding 16 right eyes (51.6%) and 15 left eyes (48.4%). Complete RPE and outer retinal atrophy was identified in 28/31 eyes (90.3%), 1 eye showed incomplete RPE and outer retinal atrophy (3.2%), and 2 eyes had no atrophy (6.5%); the latter 3 were retained in the overall cohort but excluded from atrophy subgroup analyses. Foveal involvement by atrophy was present in 14/28 GA eyes (50.0%).

**Table 1:** Demographic and Clinical Characteristics of the Study Population

Characteristic	Value
Patients, n	19
Eyes, n	31
Female, n (%)	10 (52.6)
Male, n (%)	9 (47.4)
Age, mean ± SD, years	85.6 ± 5.5
Age range, years	69–96
Right eyes, n (%)	16 (51.6)
Left eyes, n (%)	15 (48.4)
Both eyes included, n participants	12

SD = standard deviation.

Persistent BLamD was the most prevalent phenotype, identified in 28/31 eyes (90.3%), followed by scalloped BLamD and IHRF, both present in 21/31 eyes (67.7%), and spiky RPE in 18/31 (58.1%). Outer retinal corrugations were detected in 4/31 eyes (12.9%); 27/31 (87.1%) exhibited ≥2 concurrent features.

At least one additional finding was identified in 12/31 eyes (38.7%). Hypocore drusen were the most prevalent, present in 8/31 eyes (25.8%), with soft drusen in 4/31 (12.9%). Subretinal drusenoid deposits were present in 31/31 eyes (100%) (Table 2).

**Table 2:** Atrophy Status and Overall OCT Findings in the Study Cohort

Variable	Eyes, n/N (%)
cRORA	28/31 (90.3)
iRORA	1/31 (3.2)
No atrophy	2/31 (6.5)
Foveal involvement among GA eyes	14/28 (50.0)
Persistent BLamD	28/31 (90.3)
Scalloped BLamD	21/31 (67.7)
IHRF	21/31 (67.7)
Spiky RPE	18/31 (58.1)
Outer retinal corrugations	4/31 (12.9)
≥2 concurrent RPE–BLamD complex abnormalities	27/31 (87.1)
≥1 additional finding	12/31 (38.7)
HDC	8/31 (25.8)
Soft drusen	4/31 (12.9)
SDD	31/31 (100)

BLamD = basal laminar deposit; cRORA = complete retinal pigment epithelium and outer retinal atrophy; GA = geographic atrophy; HDC = hypocore drusen; IHRF = intraretinal hyperreflective foci; iRORA = incomplete retinal pigment epithelium and outer retinal atrophy; RPE = retinal pigment epithelium; SDD = subretinal drusenoid deposit.

### BLamD Groups

Eyes with scalloped BLamD (21/31, 67.7%) showed the following concurrent structural abnormalities: persistent sub-RPE material in 18/21 eyes (85.7%), spiky RPE in 12/21 eyes (57.1%), IHRF in 14/21 eyes (66.7%), and outer retinal corrugations in 2/21 eyes (9.5%). Eyes without scalloped BLamD (10/31, 32.3%) showed persistent BLamD in 10/10 eyes (100%), spiky RPE in 6/10 eyes (60.0%), IHRF in 7/10 eyes (70.0%), and outer retinal corrugations in 2/10 eyes (20.0%).

Persistent BLamD was present in 28/31 eyes (90.3%) and absent in 3 (9.7%). Eyes without persistent BLamD showed universal co-occurrence of scalloped BLamD (3/3, 100%), spiky RPE (3/3, 100%), and IHRF (3/3, 100%), with no outer retinal corrugations observed. In eyes with persistent BLamD, scalloped BLamD was present in 18/28 eyes (64.3%), spiky RPE in 15/28 eyes (53.6%), IHRF in 18/28 eyes (64.3%), and outer retinal corrugations in 4/28 eyes (14.3%).

### RPE Morphologic Changes Group

Spiky RPE was present in 18/31 eyes (58.1%) and absent in 13 (41.9%). Eyes with spiky RPE showed persistent BLamD in 15/18 (83.3%), scalloped BLamD in 12/18 (66.7%), and IHRF in 14/18 (77.8%). Notably, outer retinal corrugations were entirely absent in eyes with spiky RPE (0/18, 0%) and present exclusively in eyes without spiky RPE (4/13, 30.8%). Eyes without spiky RPE showed persistent BLamD in 13/13 (100%), scalloped BLamD in 9/13 (69.2%), and IHRF in 7/13 (53.8%).

### Foveal Involvement Group

The 28 GA eyes were stratified by foveal status. Foveal involvement was present in 14/28 eyes (50.0%) and absent in 14/28 eyes (50.0%). Eyes with foveal involvement showed higher prevalence of scalloped BLamD (10/14, 71.4%) but lower prevalences of spiky RPE (6/14, 42.9%) and IHRF (7/14, 50.0%) compared with fovea-spared eyes, which demonstrated scalloped BLamD in 8/14 eyes (57.1%), spiky RPE in 9/14 eyes (64.3%), and IHRF in 11/14 eyes (78.6%). Persistent BLamD was universal in both groups (14/14, 100% each). Outer retinal corrugations were equally distributed (2/14, 14.3% each).

## Discussion

In this study we used 3-μm axial resolution high-resolution OCT to characterize BLamD-related abnormalities in eyes with AMD. Our goal was to define the in vivo morphologic spectrum of BLamD where histologic complexity is greatest and conventional imaging remains limited. The main findings were the high prevalence of persistent BLamD, the frequent detection of scalloped configurations, IHRF and spiky RPE changes, and the coexistence of BLamD-related signatures with other AMD features. These observations demonstrate that OCT-visible BLamD is a clinically recognizable structural feature that frequently co-occurs with RPE abnormalities in advanced AMD. However, the cross-sectional design does not establish whether BLamD influences RPE remodeling, shapes the development of GA, or represents a consequence of the degenerative process.

The morphologic subtypes of BLamD observed in our cohort reflect the histopathologic taxonomy established by Sarks et al^3^^,^^10^^,^^11^ and extended to the OCT era. Sarks et al^3^ defined a grading system in which the presence of continuous macular BLamD marks the histological threshold of AMD.^3^ Van der Schaft et al^7^^,^^28^ established the molecular basis of this material, demonstrating that BLamD is located between the RPE plasma membrane and its basement membrane, with an ultrastructure consistent with excessive basement membrane secretion and consisting primarily of type IV collagen, heparan sulfate proteoglycans, and laminin. The quantitative OCT-histologic correlation showed that the RPE–BLamD complex thickens progressively across AMD stages (from 13.7 μm in normal to 17.4 μm in GA) driven by BLamD accumulation rather than RPE thickening.^1^^,^^4^^,^^20^ Within our cohort, the near-universal presence of persistent BLamD (90.3%) aligns with the histologic observation that persistent BLamD represents the predominant BLamD form in eyes with GA.

All 3 eyes without OCT-visible persistent BLamD demonstrated scalloped BLamD, spiky RPE, and IHRF. Given the very small size of this subgroup, this co-occurrence should be regarded as a preliminary descriptive observation and cannot be used to define a distinct, active, or compacted remodeling state. Spiky RPE and IHRF were observed in eyes both with and without scalloped BLamD. Outer retinal corrugations were uncommon and were identified in only 4 eyes. These cross-sectional distributions, however, cannot definitely establish whether these features belong to a common remodeling pathway or whether outer retinal corrugations represent a later disease configuration. Previous histopathologic studies provide a framework in which persistent BLamD may correspond to residual extracellular material after RPE loss, whereas scalloped BLamD may represent a more organized configuration of the deposit. In the present study, however, these interpretations remain histology-informed hypotheses because temporal sequence and tissue composition were not directly evaluated. Although all 3 eyes without persistent BLamD demonstrated scalloped BLamD, spiky RPE, and IHRF, and all 4 eyes with outer retinal corrugations lacked spiky RPE, these small-subgroup observations are preliminary and do not establish a distinct remodeling state, mutual exclusivity, or temporal sequence.

A distinctive feature of our cohort is the advanced age at presentation (mean 85.6 ± 5.5 years, range 69–96), which stands in sharp contrast to the clinical profile of EMAP, which is characterized by earlier onset and a distinctive natural history, often including night blindness followed by severe central vision loss, features that differentiate it clinically from late AMD. In the recently described high-resolution OCT EMAP cohort, patients were substantially younger than those in our AMD series with a mean age of 61.7 ± 3.9 years, yet BLamD-related abnormalities were similarly frequent: persistent BLamD was identified in 81.3% of eyes, scalloped BLamD in 64.6% of eyes, and IHRF in 56.3% of eyes.^19^ These observations are relevant to the interpretation of our AMD findings. In our older AMD cohort with prevalent GA, persistent BLamD was even more frequent, and scalloped configurations were also common, supporting the idea that the same elementary BLamD-related morphologies may be encountered across different macular atrophy phenotypes. Thus, age should not be interpreted only as an epidemiologic distinction between EMAP and AMD. The presence of similar BLamD-related OCT morphologies in AMD and EMAP indicates phenotypic overlap; however, comparisons between these separately selected cross-sectional cohorts cannot establish a shared biological substrate, common upstream or downstream mechanisms, or differences in progression rate. In this view, EMAP and AMD may share a common structural substratum at the level of the RPE–BLamD–BrM complex while differing in the upstream mechanisms and rate of progression that drive lesion development and persistence.^42^ The particularly close association between scalloped BLamD and RPE abnormalities in EMAP further supports our interpretation of scalloping in AMD as a marker of active RPE distress and extracellular matrix remodeling, rather than as an inert residual deposit.

Beyond the primary RPE–BLamD abnormalities, the presence of hypocore drusen, soft drusen, SDD, and neovascularization in a subset of eyes supports the view that BLamD-related remodeling can coexist with other AMD deposit pathways rather than define an isolated phenotype.^4^^,^^19^^,^^41^^,^^43^, ^44^, ^45^ This is consistent with the known overlap between atrophic and neovascular AMD and with the association of SDD with GA. Thus, in our cohort, these additional findings likely reflect the broader AMD microenvironment in which BLamD accumulates, persists, and interacts with lipid-rich deposits.

Spiky RPE and IHRF were numerically more frequent in foveal-sparing than in foveal-involved eyes. Because the study was cross-sectional and did not include serial imaging or measurements of lesion growth, this difference cannot establish that foveal-sparing eyes represent an earlier disease stage or have greater ongoing perilesional activity. Geographic atrophy lesions tend to expand more rapidly toward the periphery than toward the fovea, with a 2.8-fold faster peripheral rate demonstrated by one of the Fundus Autofluorescence in Age-Related Macular Degeneration studies suggesting that foveal-sparing eyes represent an earlier centripetal phase with greater ongoing perilesional activity.^46^ The morphology termed spiky RPE in this study may be considered in the context of previous histopathologic descriptions of irregular, sloughed, heaped, and multilayered RPE near the border of atrophy. Nevertheless, structural OCT alone cannot determine whether spiky RPE represents an active process, a specific cellular phenotype, or a particular temporal stage of degeneration.^11^ Previous histopathologic studies of the GA transition described cells of irregular shape and pigmentation, sloughed and heaped cells, and RPE-derived material within the neurosensory retina.^3^^,^^7^^,^^13^^,^^30^^,^^47^ The co-occurrence of spiky RPE with IHRF supports the interpretation that dysmorphic RPE cells at the active border simultaneously undergo anterior migration: abnormal RPE cells corresponding to IHRF are molecularly transdifferentiated, becoming negative for retinoid markers and positive for immune markers CD68 and CD163, confirming true phenotypic transformation rather than passive displacement.^29^^,^^30^ We therefore consider the co-occurrence of spiky RPE and IHRF to be a hypothesis-generating perilesional imaging pattern. Longitudinal OCT–histology correlation will be required to determine whether these features identify biologically active RPE remodeling, predict lesion expansion, or represent a specific stage of degeneration.

The equal distribution between foveal and extrafoveal GA (50% each) at a mean age of 85.6 years carries important clinical implications both for disease characterization and for treatment candidacy. Epidemiologically, GA most commonly originates in the perifoveal region, sparing the foveal center, and progresses both outward toward the periphery and inward toward the fovea at rates ranging from 0.53 to 2.6 mm^2^/year, with consistently faster peripheral expansion, meaning that foveal sparing is the expected early configuration of GA rather than an exceptional finding.^48^, ^49^, ^50^ Fragiotta et al reported a longitudinal reduction in the fovea-spared area in eyes with a BLamD-associated double-layer sign. Although this previous observation suggests that BLamD-related imaging findings may be associated with centripetal atrophy progression, it does not definitely establish BLamD as the causal driver. The present cross-sectional study did not evaluate atrophy progression and cannot determine whether BLamD contributes directly to foveal encroachment.^36^ Although the present study does not establish treatment relevance, BLamD phenotypes could be evaluated as candidate stratification or monitoring biomarkers in future longitudinal studies of treated and untreated eyes with GA.^51^, ^52^, ^53^ While these agents do not restore lost vision, their efficacy in slowing progression makes the identification of foveal-sparing eyes with BLamD accumulation a clinically distinct phenotype.

This study has several limitations. First, its cross-sectional design precludes assessment of temporal sequence or causality; therefore, we cannot determine whether BLamD phenotypes, spiky RPE, or IHRF precede foveal involvement, or whether loss of OCT-visible persistent BLamD represents a terminal stage or a distinct phenotype. Second, the small sample size limits subgroup analysis, particularly for eyes without persistent BLamD, and all phenotypic comparisons should be considered hypothesis-generating. Third, grading was qualitative, without quantitative measurements of BLamD thickness, RPE–BrM complex dimensions, outer retinal thickness, or atrophy area, which limits risk stratification and comparison with progression models. Finally, this was a single-center, predominantly elderly cohort, which may limit generalizability to earlier-stage or more diverse AMD populations. Moreover, visual acuity data were not available for systematic analysis, limiting assessment of the functional relevance of the observed structural phenotypes; future longitudinal studies should evaluate their relationship with visual acuity and foveal status.

In conclusion, high-resolution OCT enables detailed characterization of BLamD and associated RPE morphologic features in AMD. Persistent and scalloped BLamD, spiky RPE, IHRF, and outer retinal corrugations showed variable patterns of co-occurrence; however, the cross-sectional design does not establish their temporal sequence, progression, or causal relationships. Any proposed continuum should therefore be considered a histology-informed, hypothesis-generating model. Longitudinal eye-tracked imaging and quantitative spatial analyses are needed to determine whether these features represent sequential, alternative, or independent configurations and whether they have prognostic significance for atrophy progression.

## References

[1] Curcio C.A., Millican C.L. (1999). Basal linear deposit and large drusen are specific for early age-related maculopathy. Arch Ophthalmol.

[2] Green W.R., Enger C. (1993). Age-related macular degeneration histopathologic studies. The 1992 Lorenz E. Zimmerman Lecture. Ophthalmology.

[3] Sarks S., Cherepanoff S., Killingsworth M., Sarks J. (2007). Relationship of basal laminar deposit and membranous debris to the clinical presentation of early age-related macular degeneration. Investig Opthalmology Vis Sci.

[4] Sura A.A., Chen L., Messinger J.D. (2020). Measuring the contributions of basal laminar deposit and bruch's membrane in age-related macular degeneration. Investig Opthalmology Vis Sci.

[5] Forte P., Ferro Desideri L., Nassisi M. (2026). Drusen-specific dark adaptation profiles in intermediate age-related macular degeneration. Int J Retina Vitr.

[6] Huang J., Nittala M.G., Corradetti G. (2026). Spatial distribution of cuticular drusen and its association with category-specific progression risk in intermediate AMD. Eye.

[7] Van Der Schaft T.L., De Bruijnz W.C., Mooy C.M., De Jong P.T.V.M. (1993). Basal laminar deposit in the aging peripheral human retina. Graefes Arch Clin Exp Ophthalmol.

[8] Quarta A., Feo A., Corradetti G. (2025). Double layer sign in chorioretinal diseases: clinical significance and implications from multimodal imaging. Surv Ophthalmol.

[9] Feo A., Ramtohul P., Govetto A. (2025). En face OCT: breakthroughs in understanding the pathoanatomy of retinal disease and clinical applications. Prog Retin Eye Res.

[10] Sarks S.H. (1976). Ageing and degeneration in the macular region: a clinico-pathological study. Br J Ophthalmol.

[11] Sarks J.P., Sarks S.H., Killingsworth M.C. (1988). Evolution of geographic atrophy of the retinal pigment epithelium. Eye.

[12] Ooto S., Vongkulsiri S., Sato T. (2014). Outer retinal corrugations in age-related macular degeneration. JAMA Ophthalmol.

[13] Dolz-Marco R., Balaratnasingam C., Messinger J.D. (2018). The border of macular atrophy in age-related macular degeneration: a clinicopathologic correlation. Am J Ophthalmol.

[14] Quarta A., Alhelaly M., Romano F. (2026). High-Resolution OCT profiling of bruch's membrane in extensive macular atrophy with pseudodrusen-like appearance and geographic atrophy. Invest Ophthalmol Vis Sci.

[15] Quarta A., Abbasgholizadeh R., Soylu C., Sadda S.R. (2026). Evolution of Intraretinal Sclerochoroidal Vessels over two years follow-up. Retina.

[16] Quarta A., Soylu C., Sadda S.R. (2026). High-resolution OCT of ILM hyperconvolution associated with epiretinal membrane. Can J Ophthalmol J Can Ophtalmol.

[17] Won J., Takahashi H., Ploner S.B. (2024). Topographic measurement of the subretinal pigment epithelium space in normal aging and age-related macular degeneration using high-resolution OCT. Invest Ophthalmol Vis Sci.

[18] Alhelaly M., Soylu C., Corradetti G. (2025). In vivo characterization of bruch's membrane breaks in neovascular age-related macular degeneration using high-resolution optical coherence tomography. Invest Ophthalmol Vis Sci.

[19] Quarta A., Romano F., Alhelaly M. (2026). High-Resolution OCT of presumed basal laminar deposit and RPE abnormalities in extensive macular atrophy with pseudodrusen-like appearance. Ophthalmol Retina.

[20] Curcio C.A., Johnson M., Huang J.D., Rudolf M. (2009). Aging, age-related macular degeneration, and the response-to-retention of apolipoprotein B-containing lipoproteins. Prog Retin Eye Res.

[21] Chujo S., Quarta A., Corradetti G. (2026). Photoreceptor density–dependent kinetics of geographic atrophy progression. Ophthalmol Sci.

[22] Ferris F.L., Wilkinson C.P., Bird A. (2013). Clinical classification of age-related macular degeneration. Ophthalmology.

[23] Sadda S.R., Guymer R., Holz F.G. (2018). Consensus definition for atrophy associated with age-related macular degeneration on OCT. Ophthalmology.

[24] Fragiotta S., Parravano M., Sacconi R. (2022). A common finding in foveal-sparing extensive macular atrophy with pseudodrusen implicates basal laminar deposits. Retina.

[25] Curcio C.A., Johnson M., Rudolf M., Huang J.D. (2011). The oil spill in ageing Bruch membrane. Br J Ophthalmol.

[26] Hirabayashi K., Yu H.J., Wakatsuki Y. (2023). OCT risk factors for development of atrophy in eyes with intermediate age-related macular degeneration. Ophthalmol Retina.

[27] Eckardt C., Eckardt U., Groos S. (2004). Macular translocation in a patient with adult-onset foveomacular vitelliform dystrophy with light- and electron-microscopic observations on the surgically removed subfoveal tissue. Graefes Arch Clin Exp Ophthalmol.

[28] Van Der Schaft T.L. (1991). Is basal laminar deposit unique for age-related macular degeneration?. Arch Ophthalmol.

[29] Zanzottera E.C., Messinger J.D., Ach T. (2015). The Project MACULA retinal pigment epithelium grading System for histology and optical coherence tomography in age-related macular degeneration. Investig Opthalmology Vis Sci.

[30] Zanzottera E.C., Ach T., Huisingh C. (2016). Visualizing retinal pigment epithelium phenotypes in the transition to geographic atrophy in age-related macular degeneration. Retina.

[31] Boscia G., Feo A., Quarta A. (2026). Intraretinal hyperreflective foci: pathophysiology, imaging features, and clinical implications across retinal diseases. Prog Retin Eye Res.

[32] Sarraf D., Corvi F., Rosenfeld P.J. (2026). Standardizing measurement of cRORA (complete retinal pigment epithelial and outer retinal Atrophy)- CAM report 8. Ophthalmol Retina.

[33] Nassisi M., Fan W., Shi Y. (2018). Quantity of intraretinal hyperreflective foci in patients with intermediate age-related macular degeneration correlates with 1-Year progression. Investig Opthalmology Vis Sci.

[34] Verma A., Nittala M.G., Haines J.L. (2025). Spatial distribution of intra-retinal Hyper-reflective foci and impact on progression in eyes with intermediate Age-related macular degeneration. Graefes Arch Clin Exp Ophthalmol.

[35] Mahmoudi A., Manafi N., Corradetti G. (2024). Risk factors for development of hyper-reflective foci overlying drusen in eyes with intermediate age-related macular degeneration. Br J Ophthalmol.

[36] Fragiotta S., Parravano M., Sacconi R. (2025). Progression patterns in foveal-sparing geographic atrophy with double-layer sign due to neovascularization or basal laminar deposits. Invest Ophthalmol Vis Sci.

[37] Borrelli E., Barresi C., Berni A. (2024). OCT risk factors for 2-year foveal involvement in non-treated eyes with extrafoveal geographic atrophy and AMD. Graefes Arch Clin Exp Ophthalmol.

[38] Zweifel S.A., Spaide R.F., Curcio C.A. (2010). Reticular pseudodrusen are subretinal drusenoid deposits. Ophthalmology.

[39] Oncel D., Corradetti G., Wakatsuki Y. (2023). Drusen morphometrics on optical coherence tomography in eyes with age-related macular degeneration and normal aging. Graefes Arch Clin Exp Ophthalmol Albrecht Von Graefes Arch Klin Exp Ophthalmol.

[40] Mastropasqua R., Quarta A., Passamonti M. (2026). Macular pigment optical density following lutein scleral iontophoresis in intermediate age-related macular degeneration: a six-month pilot study. Br J Ophthalmol.

[41] Goh K.L., Abbott C.J., Hadoux X. (2022). Hyporeflective cores within Drusen. Ophthalmol Retina.

[42] Quarta A., Corradetti G., Romano F. (2025). Distinct profiles of choriocapillaris involvement in extensive macular atrophy with pseudodrusen-like appearance and geographic atrophy. Invest Ophthalmol Vis Sci.

[43] Spaide R.F. (2024). Pathways to geographic atrophy in nonneovascular age-related macular degeneration. Retina.

[44] Chujo S., Quarta A., Abbasgholizadeh R. (2026). Development of a conceptual, HOV-inspired spatial weighting model for quantifying geographic atrophy severity. Graefes Arch Clin Exp Ophthalmol Albrecht Von Graefes Arch Klin Exp Ophthalmol.

[45] Abbasgholizadeh R., Quarta A., Alhelaly M. (2026). Factors impacting the assessment of geographic atrophy on ultra-widefield fundus autofluorescence imaging. Graefes Arch Clin Exp Ophthalmol Albrecht Von Graefes Arch Klin Exp Ophthalmol.

[46] Holz F.G., Bindewald-Wittich A., Fleckenstein M. (2007). Progression of geographic atrophy and impact of fundus autofluorescence patterns in age-related macular degeneration. Am J Ophthalmol.

[47] Chen L., Messinger J.D., Zhang Y. (2020). Subretinal drusenoid deposit in age-related macular degeneration: histologic insights into initiation, progression to atrophy, and imaging. Retina.

[48] Li M., Dolz-Marco R., Huisingh C. (2019). Clinicopathologic correlation of geographic atrophy secondary to age-related macular degeneration. Retina.

[49] Fleckenstein M., Issa P.C., Helb H.M. (2008). High-Resolution spectral Domain-OCT imaging in geographic atrophy associated with age-related macular degeneration. Investig Opthalmology Vis Sci.

[50] Fleckenstein M., Mitchell P., Freund K.B. (2018). The progression of geographic atrophy secondary to age-related macular degeneration. Ophthalmology.

[51] Fu D.J., Bagga P., Naik G. (2024). Pegcetacoplan treatment and consensus features of geographic atrophy over 24 months. JAMA Ophthalmol.

[52] Heier J.S., Lad E.M., Holz F.G. (2023). Pegcetacoplan for the treatment of geographic atrophy secondary to age-related macular degeneration (OAKS and DERBY): two multicentre, randomised, double-masked, sham-controlled, phase 3 trials. Lancet.

[53] Khanani A.M., Danzig C.J., Heier J.S. (2026). Avacincaptad pegol for geographic atrophy secondary to age-related macular degeneration: Two-Year efficacy and safety results from the GATHER2 phase 3 trial. Ophthalmology.

