# Supplementary material for: High-Resolution OCT of Basal Laminar Deposit Phenotypes and Retinal Pigment Epithelium Morphologic Changes in Age-Related Macular Degeneration
Source: Ophthalmol Sci. 2026 Sep 23;6(12):101415. doi: 10.1016/j.xops.2026.101415 (PMC13720542; doi:10.1016/j.xops.2026.101415)

Supplementary material 1. Flow chart for the inclusion of the final cohort.

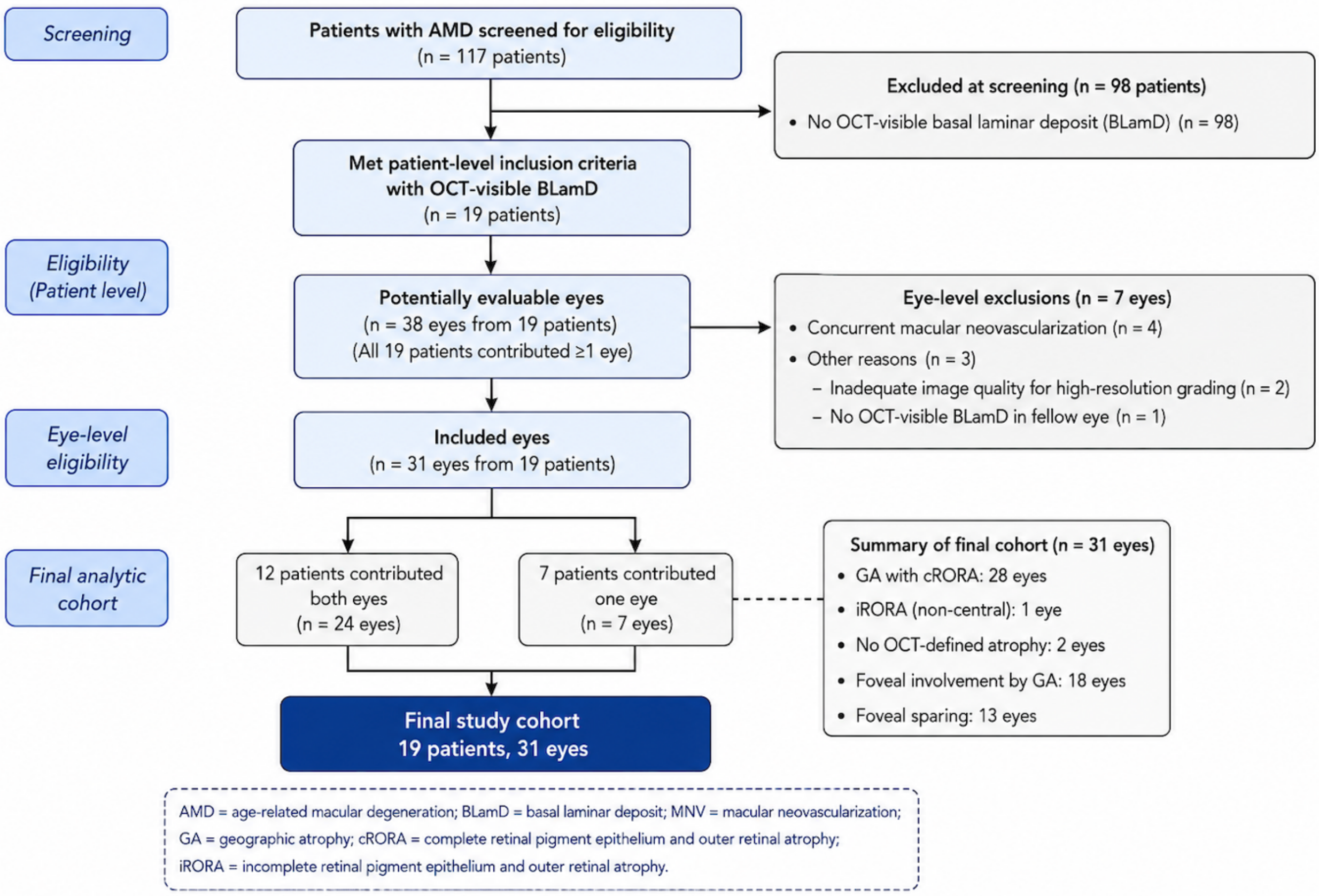

Supplement: Supplementary file 1 [file mmc1.pdf]
